# Differential influence of different dietary fatty acids on very low-density lipoprotein secretion when delivered to hepatocytes in chylomicron remnants

**DOI:** 10.1016/j.metabol.2008.09.012

**Published:** 2009-02

**Authors:** Iliana López-Soldado, Michael Avella, Kathleen M. Botham

**Affiliations:** Department of Veterinary Basic Sciences, The Royal Veterinary College, NW1 0TU London, United Kingdom

## Abstract

The influence of dietary fats carried in chylomicron remnants on the hepatic secretion of very low-density lipoprotein (VLDL) was investigated using chylomicron remnant–like particles (CRLPs) and cultured rat hepatocytes as the experimental model. Chylomicron remnant–like particles containing triacylglycerol (TG) from palm, olive, or corn (enriched in saturated, monounsaturated, or n-6 polyunsaturated fatty acids) oil, respectively, were incubated with cultured hepatocytes for 5 hours. The medium was then removed and replaced with medium without CRLPs; and the secretion of TG, cholesterol, and apolipoprotein B48 during the following 16 hours was determined. Secretion of TG into the *d* less than 1.050–g/mL fraction containing VLDL was unaffected by olive CRLPs, but was significantly increased in cells exposed to palm or corn CRLPs in comparison with both olive CRLPs and control incubations without CRLPs. Secretion of apolipoprotein B48, however, was not changed by any of the CRLP types. Apolipoprotein B messenger RNA levels were decreased by olive and corn CRLPs, and 3-hydroxy-3-methylglutaryl coenzyme A reductase messenger RNA abundance was increased by palm CRLPs; but expression of other genes involved in the regulation of VLDL secretion was unaffected. These findings demonstrate that CRLPs enriched in saturated fatty acids or n-6 polyunsaturated fatty acids increase the secretion of TG in VLDL, possibly because of the secretion of larger particles, whereas those enriched in monounsaturated fatty acids have no effect. Thus, different dietary fats have differential effects on VLDL secretion directly when delivered to the liver in chylomicron remnants.

## 1 Introduction

It is well established that increased plasma levels of cholesterol and triacylglycerol (TG) are associated with higher risk of atherosclerosis [1,2]. The detrimental effects of hypercholesterolemia are well known, and hypertriglyceridemia is now accepted as an independent risk factor [3]. Many epidemiologic, experimental, and clinical studies in human and animal have shown that the amounts and type of fat in the diet influence plasma lipid levels and thereby influence atherosclerosis risk [4-6]. Saturated fatty acids (SFA) are the most potent cholesterol-raising dietary component, whereas monounsaturated (MUFA) and n-6 polyunsaturated fatty acids (PUFA) are cholesterol lowering [7,8]; and modulation of circulating low-density lipoprotein (LDL) cholesterol levels is the main mechanism by which SFA, MUFA, and n-6 PUFA exert their effects [9-11]. n-3 PUFA, on the other hand, benefit coronary heart disease risk by lowering plasma TG through decreased very low-density lipoprotein (VLDL) concentrations [12,13].

Lipids, including fats and cholesterol from the diet, are absorbed from the intestine in chylomicrons; and these large TG-rich lipoproteins are secreted into lymph and pass into the blood via the thoracic duct. They then undergo rapid lipolysis by lipoprotein lipase in extrahepatic capillary beds, removing some of the TG and leaving smaller chylomicron remnants (CMR) that deliver the remaining dietary lipids to the liver [14,15]. Chylomicron remnants contain apolipoprotein (apo) B48, which is secreted as an integral part of the parent chylomicrons, and apo E, which is acquired in the circulation, and are taken up by the liver by apo E–dependent receptor–mediated processes involving the LDL receptor and the LDL receptor–related protein [15,16].

The liver has a central role in the regulation of cholesterol homeostasis, as excretion via the bile, either unchanged or after conversion to bile acids, as it is the only route by which cholesterol may be removed from the body in significant quantities. At the same time, it is a major site for the synthesis of cholesterol for secretion into blood via VLDL, which is converted to LDL in the circulation [17,18]. Thus, cholesterol taken up in the plasma lipoproteins may be secreted into bile; or alternatively, it may be returned to the blood in VLDL. The balance between these opposing hepatic functions, therefore, plays a large part in determining blood cholesterol levels.

Feeding studies in which animals are kept on fat-enriched diets have demonstrated that saturated and unsaturated fats have differential effects on hepatic VLDL secretion. The hypotriacylglycerolemic effect of dietary n-3 PUFA is related to inhibition of hepatic VLDL assembly and secretion [19-21], an effect that is not observed with dietary n-6 PUFA [22]; and Xie et al [23] have reported that hepatic secretion of lipoprotein cholesterol is increased in fat-fed mice, with MUFA having the greatest effect, followed by n-6 PUFA and then SFA. This type of study, however, cannot distinguish between the direct effects of dietary fats when delivered to the liver in CMR and the effects that arise subsequently as a result of changes on the fatty acid composition of the tissues or other lipoproteins such as LDL. In other work, the direct addition of different nonesterified fatty acids (NEFA) to cultured hepatocytes has been found to modulate VLDL secretion, with SFA, MUFA, and n-6 PUFA causing an increase and n-3 PUFA causing a decrease [24-26]. Fatty acids from the diet, however, reach the liver in TG in CMR rather than as NEFA; and Brown et al [27] have reported important differences in the effects of n-3 PUFA on VLDL secretion, depending on whether the fatty acid was given in the diet in fish oil or added to hepatocyte cultures as NEFA. Previous work in our laboratory has demonstrated that CMR derived from fish oil enriched in n-3 PUFA directly suppress the secretion of TG, cholesterol, and apo B48 in VLDL in cultured rat liver cells, whereas particles enriched in n-6 PUFA had no effect [28]. Little is known, however, about the effects of CMR enriched in MUFA or SFA on VLDL secretion.

The aim of the present work is to compare the effects of SFA, MUFA, and n-6 PUFA from the diet in the direct modulation of the synthesis and secretion of VLDL when delivered to the liver in CMR. Cultured primary rat hepatocytes and chylomicron remnant–like particles (CRLPs) containing TG derived from palm, olive, or corn oil were used as the experimental model; and the secretion of TG, cholesterol, and apo B48 into the medium and the expression of messenger RNA (mRNA) for various genes that have an important role in the regulation of hepatic VLDL synthesis, assembly, and secretion were determined.

## 2 Materials and methods

### 2.1 Animal and materials

Male Wistar rats (300-350 g body mass) were fed a standard pellet diet, housed under conditions of constant day length (12 hours), and allowed access to food and water ad libitum. All animal experiments were approved by the Royal Veterinary College Ethics and Welfare Committee.

Ketamine and Rompun were obtained from Fort Dodge Animal Health LTD (Southampton, United Kingdom) and Bayer (Wuppertal, Germany), respectively. Collagenase, bovine serum albumin (BSA), Percoll (GE Healthcare Bio-Sciences, Uppsala, Sweden), RPMI 1640, trypan blue, phospholipids, cholesterol, cholesteryl oleate, gentamicin, nystatin, insulin, pyruvate, dexamethasone, penicillin, and streptomycin were supplied by Sigma (Poole, Dorset, United Kingdom). Fetal bovine serum (heat inactivated) was obtain from Gibco (Paisley, United Kingdom). Palm oil (KTC Edibles, Wednesbury, United Kingdom), extra virgin olive oil (Bertolli; Unilever Foods, Crawley, Surrey, United Kingdom), and corn oil (Mazola; Bestfoods, Esher, United Kingdom) were purchased from domestic suppliers.

### 2.2 Preparation of CRLPs

Triacylglycerols for CRLP preparation were isolated from palm, olive, and corn oils as follows: 1.5 mL of each oil was added to 30 mL chloroform/methanol (2:1, vol/vol) and 0.88% KCl (40% total volume), mixed, and left at 4°C overnight. The upper aqueous phase was then removed, and TGs were isolated from the chloroform phase by thin layer chromatography (hexane/diethyl ether/formic acid, 80:20:2, vol/vol/vol). The band corresponding to TG was collected, resuspended in chloroform, and centrifuged twice at 1200*g* for 20 minutes (4°C) (MSE Mistral 3000i centrifuge; MSE, London, United Kingdom) in a swing out rotor to remove the silica gel. The chloroform supernatant was collected after each centrifugation and kept under argon at −20°C until required.

The CRLPs were prepared by sonication (power setting, 50 W; 20 minutes; at 56°C) of a lipid mixture containing 70% TG extracted from oils as above, 2% cholesterol, 5% cholesteryl ester (CE), and 25% phospholipids in 0.9% (wt/vol) NaCl in tricine buffer (20 mmol/L, pH 7.4). The resulting emulsion was brought to a density of 1.21 g/mL with KBr, layered under a stepwise density gradient as described by Diard et al [29], and centrifuged at 17 000*g* for 20 minutes at 20°C (Beckman Optima L-80 centrifuge; Beckman Coulter, High Wycombe, United Kingdom) in an SW40Ti swing out rotor. The upper layer of grossly emulsified lipids was then removed and replaced with an equal volume of NaCl solution (*d* = 1.020 g/mL), and tubes were centrifuged at 70 000*g* for 1 hour (20°C) in a SW40Ti swing out rotor. For apo E binding, lipid particles harvested from the top layer were incubated (2:1, vol/vol) with the *d* greater than 1.020–g/mL fraction of rat plasma (Charles River, London, United Kingdom) prepared by ultracentrifugation and dialyzed before use as described previously [30] at 37°C with shaking for 4 hours. The CRLPs were then isolated by ultracentrifugation at *d* = 1.006 g/mL (120 000*g* for 12 hours at 4°C), harvested from the top layer, purified by a second centrifugation at the same density (202 000*g* for 4 hours at 4°C), and stored at 4°C under argon until required. All preparations were used within 1 week.

### 2.3 Preparation and culture of hepatocytes

Hepatocytes were isolated from rat livers by perfusion with collagenase, as described previously [31]. The cells were resuspended in RPMI 1640 containing sodium bicarbonate (2 g/L), penicillin (100 000 U/L), streptomycin (100 mg/L), gentamicin (50 mg/L), glucose (2 g/L), pyruvate (110 mg/L), dexamethasone (0.1 *μ*mol/L), and nystatin (20 000 U/L) (supplemented RPMI) and applied to a 0% to 70% (vol/vol) Percoll gradient to separate the viable from the nonviable cells. The viable cells were washed twice with supplemented RPMI containing BSA (2%), and finally resuspended in supplemented RPMI containing 10% (vol/vol) fetal bovine serum and insulin (4 mg/L). Cell viability, as assessed by trypan blue exclusion, was routinely greater than 90%. The cells were cultured in Primaria-coated plastic Petri dishes (BD, Franklin Lakes, NJ) at 37°C in an atmosphere of air/CO_2_ (19:1), as described by Isusi et al [32]. After adhesion of the cells to the dishes, the medium was then removed and replaced with supplemented RPMI medium containing 60 *μ*g/L insulin, but without fetal bovine serum. The cultures were incubated in the presence of CRLPs derived from corn, olive, or palm oil (separate CRLP preparations of each type were used for each hepatocyte preparation), or with an equal volume of culture medium, at 37°C in air/CO_2_ (19:1) for 5 hours. The medium was then removed, the cells were washed 3 times with supplemented RPMI, fresh medium without CRLPs was added, and the incubation was continued for 16 hours. The medium was then collected and either extracted with chloroform/methanol (2:1, vol/vol) or centrifuged at 104 000*g* for 16 hours at *d* less than 1.050 g/mL, and the top fraction containing VLDL was obtained by tube slicing. A density of *d* less than 1.050 g/mL was chosen to exclude high-density lipoproteins, but to include any intermediate-density lipoproteins or LDL that may have been formed by the action on VLDL of lipases secreted by the cells [33]. The cells were harvested from the dishes, washed twice with PBS, and disrupted by sonication for 5 seconds (50 W, power setting); a sample was taken for protein determination; and lipids were extracted with chloroform/methanol (2:1 vol/vol) for lipid assays.

For mRNA determination, after adhesion of the cells to the dishes, the medium was removed and replaced with supplemented RPMI containing 60 *μ*g/L insulin, but without fetal bovine serum. The medium was changed the next morning; and the cultures were incubated in the presence of CRLPs derived from corn, olive, and palm for 5 and 16 hours. The total RNA was then extracted from the cells as described below.

### 2.4 Analytical methods

The TG and total cholesterol (TC) or unesterified cholesterol (UC) contents in CRLPs, cells, medium, or VLDL extracts were determined by enzymatic reagent kits (Alpha Laboratories, Eastleigh, United Kingdom); and the CE content was calculated by subtracting the value for UC from that for TC. Cell protein content was measured by the method of Lowry et al [34] using BSA as a standard. The relative apo E content of the different types of CRLPs was assessed using sodium dodecyl sulfate polyacrylamide gel electrophoresis (SDS-PAGE). The gels were stained with Coomassie blue, and the bands were quantified by optical density volume analysis. Apolipoprotein B48 in the medium was separated by SDS-PAGE and quantified by Western blotting coupled to enhanced chemiluminescence (ECL) analysis [35] after preparation of the samples, as described by Mindham and Mayes [36].

The relative abundance of mRNA transcripts for the genes under study was determined by quantitative polymerase chain reaction (qPCR). Total RNA was extracted from hepatocytes using a GenElute Mammalian Total RNA Kit (Sigma, St Poole, Dorset, United Kingdom) according to the protocol provided by the manufacturer. First-strand complementary DNA was synthesized using Reverse Transcription System (Promega, Madison, WI). The qPCR was performed in a LightCycler system (DNA Engine Opticon 2, MJ Research, Waltham, MA) using SYBR Green fluorescence. The PCR protocol involved a denaturation program (94°C for 2 minutes), followed by amplification and quantification program repeated 40 times (94°C for 15 seconds, 59°C for 1 minute, 72°C for 1 minute), and finally a melting curve program (65°C-95°C with a heating rate of 0.2°C/s). The forward and reverse oligonucleotide primers used for the genes tested are shown in Table 1. The Ct values were determined by automated threshold analysis by using Opticon Monitor 3.1 software. The relative quantification of gene expression was calculated by the method of Pfaffl [37]. Glyceraldehyde-3-phosphate dehydrogenase (GAPDH) was used as a housekeeping gene, and all results were normalized to the abundance of GAPDH mRNA.

### 2.5 Statistical analysis

Data were analyzed using SPSS version 14 (SPSS, Chicago, IL). Statistical significance was calculated using 1-or 2-way analysis of variance and by Student paired *t* test or Bonferroni post hoc test, respectively.

## 3 Results

### 3.1 Characteristics of CRLPs

The content of TG, TC, UC, and EC and the TG/TC ratio in CRLPs containing TG from palm (palm CRLPs), olive (olive CRLPs), and corn (corn CRLPs) oils are shown in Table 2. There were no significant differences in the TG, TC, UC, and CE concentrations and, more importantly, in the TG/TC ratio between palm, olive, and corn CRLPs. Thus, although the CRLPs were added to the cell incubations according to the TG concentration, the amount of TC present was also similar in experiments with the different types of particles. Previous studies in our laboratory have shown that CRLPs prepared with TG from palm, olive, or corn oil contain relatively high concentrations of SFA (particularly palmitic acid [16:0]), MUFA (mainly oleic acid [18:1, n-9]), or n-6 PUFA (mainly linoleic acid [18:2, n-6]), respectively, and that their fatty acid composition generally resembles that of the parent oils [38,39].

**Fig. 1:**
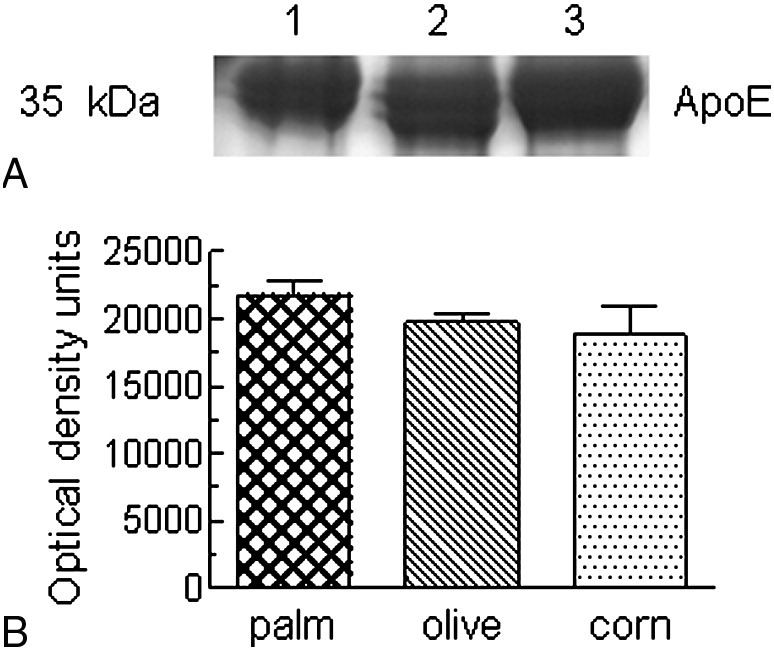
Chylomicron remnant–like particles were prepared as described in “Materials and methods,” and the apo E content was assessed by SDS-PAGE. A, Apolipoprotein E bands in a typical experiment: Lane 1, palm CRLPs; lane 2, olive CRLPs; lane 3, corn CRLPs. B, Quantification by optical density volume analysis. Data shown are the mean ± SEM from 3 separate preparations.

The apo E content of the palm, olive, and corn CRLPs was assessed by SDS-PAGE; and the results of a typical experiment are shown in Fig. 1A. Optical density volume analysis of the apo E bands showed that there were no significant differences between the 3 CRLP types (Fig. 1B).

### 3.2 Effect of CRLPs derived from palm, olive, or corn oil on the secretion of lipids by cultured rat hepatocytes

Cultured rat hepatocytes were incubated with or without CRLPs derived from palm, olive, and corn oil (0.3 *μ*mol TG per milliliter) for 5 hours. The medium was then removed, and the secretion of TG and cholesterol into the medium or into the *d* less than 1.050–g/mL fraction during a further 16 hours of incubation was determined.

**Fig. 2:**
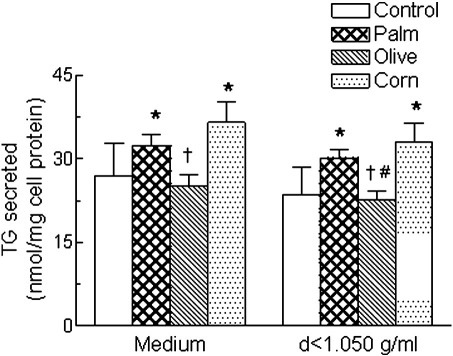
Hepatocytes (6 × 10^6^) were incubated with or without CRLPs derived from palm, olive, or corn oil (0.3 *μ*mol TG per milliliter) for 5 hours; the medium was then removed and replaced with fresh medium without CRLPs; and the incubation was continued for a further 16 hours. After this time, the medium was collected; and the content of TG in the medium or in the *d* less than 1.050–g/mL fraction was determined. Data are expressed as nanomoles per milligram cell protein and are the mean ± SEM from 8 separate hepatocyte preparations, each performed in duplicate. Significant limits: *P < .05 vs control (incubation without CRLPs); ^†^*P* < .05 vs corn CRLPs; ^#^*P* < .05 vs palm CRLPs.

The results (Fig. 2) showed that the amount of TG secreted into the medium was significantly increased in hepatocytes treated with palm or corn, but not olive, CRLPs in comparison with cells incubated without CRLPs. Furthermore, TG secretion was lower in the presence of olive as compared with corn and palm CRLPs, although this change was significant only for corn CRLPs. No significant changes were detected in the secretion of TC, UC, or CE into the medium by cells incubated with or without the different types of CRLPs, although secretion tended to be lower with olive CRLPs than with palm or corn CRLPs in all cases (data not shown).

When the medium of the cells was fractionated by ultracentrifugation, 87% to 95% of the TG and 70% to 80% of the cholesterol was recovered in the top fraction (*d* < 1.050 g/mL) containing VLDL. As shown in Fig. 2, secretion of TG into the *d* less than 1.050–g/mL fraction showed a similar pattern to that in the whole medium, with a significant increase observed in incubations with palm and corn CRLPs as compared with the control without CRLPs and significantly lower secretion after treatment with olive CRLPs as compared with corn CRLPs and, in this case, also palm CRLPs. There were no significant differences in the TC, UC, or CE secreted into the *d* less than 1.050–g/mL fraction (data not shown).

**Fig. 3:**
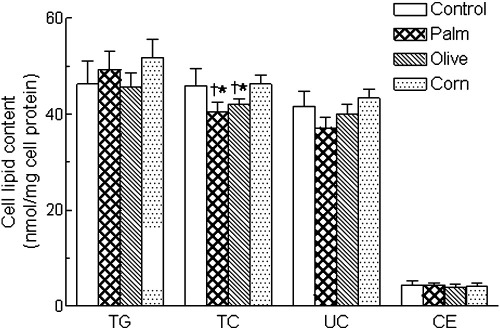
Hepatocytes (6 × 10^6^) were incubated with or without CRLPs derived from palm, olive, or corn oil (0.3 *μ*mol TG per milliliter) for 5 hours; the medium was then removed and replaced with fresh medium without CRLPs; and the incubation was continued for a further 16 hours. After this time, the cells were harvested; and their content of TG, TC, UC, and CE was determined. Data are expressed as nanomoles per milligram cell protein and are the mean ± SEM from 9 separate hepatocyte preparations, each performed in duplicate. Significant limits: *P < .05 vs control (incubation without CRLPs); ^†^*P* < .05 vs corn CRLPs.

Intracellular TG accumulation in hepatocytes was not affected by the addition of the different CRLPs (Fig. 3). In contrast, the cellular level of TC was lower in hepatocytes exposed to olive or palm, but not corn, CRLPs as compared with control incubations without CRLPs; and as a consequence of this, the TC content of the cells was significantly lower after treatment with olive or palm CRLPs rather than corn CRLPs (Fig. 3). The UC levels in the cells showed a similar pattern of changes, although the differences did not reach significance, whereas, in contrast, the CE concentrations were almost identical in all conditions. We conclude, therefore, that the changes in cell TC content are mainly due to differences in the UC levels in the cells.

### 3.3 Effect of CRLPs derived from palm, olive, or corn oil on the secretion of apo B by cultured rat hepatocytes

**Fig. 4:**
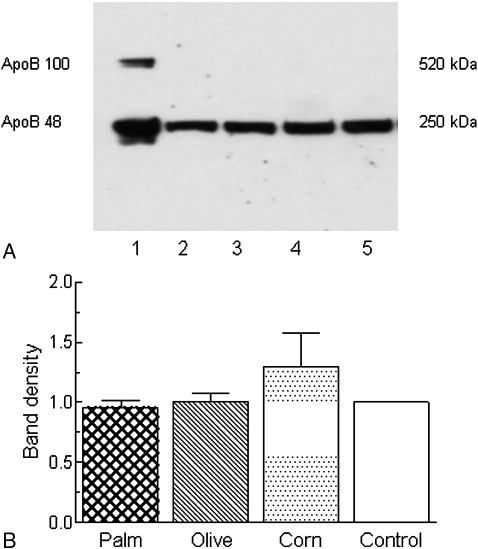
Hepatocytes (6 × 10^6^) were incubated with or without CRLPs derived from palm, olive, or corn oil (0.3 *μ*mol TG per milliliter) for 5 hours; the medium was then removed and replaced with fresh medium without CRLPs; and the incubation was continued for a further 16 hours. After this time, the medium was collected; and the content of apo B48 was determined. A, Apolipoprotein B48 bands in a typical experiment. B, Quantification by Western blotting coupled to ECL. Data are expressed as the percentage of the control in arbitrary units per milligram cell protein and are the mean ± SEM from 8 separate hepatocyte preparations, each performed in duplicate. Lane 1, positive control containing apo B48 and apo B100; lane 2, palm CRLPs; lane 3, olive CRLPs; lane 4, corn CRLPs; lane 5, without CRLPs.

The levels of apo B48 secreted into the medium after incubation of hepatocytes with or without palm, olive, or corn CRLPs were determined using SDS-PAGE and Western blotting coupled to ECL analysis [8] (Fig. 4). Visual examination of the bands suggested that the CRLPs caused no significant changes in secretion (Fig. 4A), and this was confirmed by optical density volume analysis (Fig. 4B). Concentrations of apo B48 in the *d* less than 1.050–g/mL fraction were too low to measure accurately, as were levels of apo B100 in the whole medium.

### 3.4 Effect of CRLPs derived from palm, olive, and corn oil on mRNA expression for genes related to VLDL synthesis in cultured rat hepatocytes

**Fig. 5:**
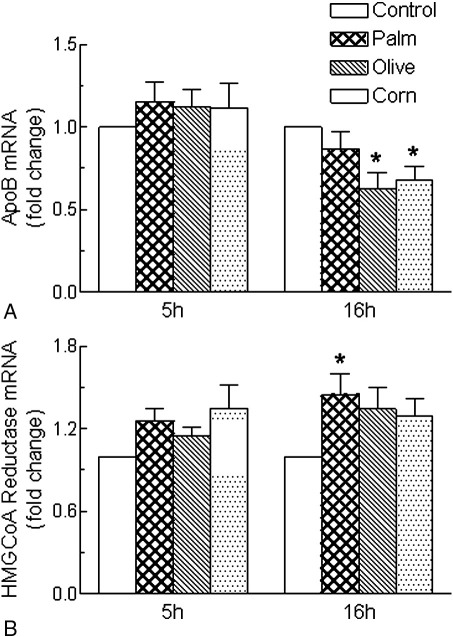
Hepatocytes (1.2 × 10^6^) were incubated with or without CRLPs derived from palm, olive, or corn oil (0.3 *μ*mol TG per milliliter) for 5 or 16 hours. The medium was then removed, the total RNA was extracted from the cells, and the relative abundance of transcripts for (A) apo B and (B) HMG-CoA reductase was determined by qPCR. Values are expressed as fold change relative to the control (hepatocytes incubated without CRLPs). Data are the mean ± SEM from 8 separate hepatocyte preparations, each performed in duplicate. *P < .05 vs control (2-way analysis of variance).

Fig. 5 shows the effects of incubation of cultured rat hepatocytes with CRLPs derived from palm, olive, and corn oil for 5 or 16 hours on the relative abundance of mRNA transcripts for apo B and 3-hydroxy-3-methylglutaryl coenzyme A reductase (HMG-CoA reductase). Results are expressed as fold change in comparison with control cells incubated without CRLPs. The expression of mRNA for apo B was not significantly changed by any of the CRLP types after incubation for 5 hours; but after 16 hours, the level of apo B mRNA was significantly decreased in cells exposed to olive and corn CRLPs as compared with those incubated without CRLPs (control) (Fig. 5A). No changes in the expression of mRNA for HMG-CoA reductase were observed after 5 hours (Fig. 5B) with the different types of CRLPs. After 16 hours, however, HMG-CoA reductase mRNA levels tended to be increased by all 3 types of particles, although this change reached significance only in cells exposed to palm CRLPs compared with those incubated without CRLPs (control) (Fig. 5B). The expression of mRNA for MTP, ACAT-2, or mitochondrial GPAT (mGPAT) in the hepatocytes was not changed after 5 or 16 hours of incubation by any of the CRLP types tested (data not shown).

## 4 Discussion

In this study, we have used cultured rat hepatocytes to investigate the direct effects of dietary SFA, MUFA, and n-6 PUFA on the hepatic VLDL secretion directly when delivered to the liver in CMR and to determine whether the mechanism of these effects involves changes in the transcription of various genes that have an important role in the regulation of hepatic VLDL synthesis, assembly, and secretion. It is important to use primary hepatocytes for this type of study because the complex liver functions needed for lipoprotein secretion are often impaired in cell lines. For this reason, the rat, which is one of the few convenient sources of primary liver cells and has been widely used as a model for VLDL secretion [17,18], was chosen for the experiments.

It is difficult to separate CMR from the blood from other lipoproteins of a similar density that are present postprandially, such as chylomicrons and VLDL, by the conventional ultracentrifugation methods used for lipoprotein preparation. In previous studies with rats, we have used chylomicrons isolated from the chyle collected via cannulation of the thoracic duct after a fat meal to prepare CMR by infusion of the chylomicrons into animals that have been functionally eviscerated followed by isolation of the remnants from the blood [28,38,40]. This procedure, however, requires large numbers of rats; and more recently, we have developed an alternative approach using CRLPs containing apo E to minimize animal use [30,41]. These model particles have other advantages in that they can be applied to any species, as the appropriate apo E can be incorporated, and that their lipid content can be easily manipulated. In previous work [42], we have found that the particles have a diameter similar to that of human CMR (50-150 nm) [43]. In addition, the lipid composition (TG and cholesterol content and the TG/TC molar ratio) is similar to that of physiologic remnants [14]. The CRLPs used, therefore, resemble physiologic CMR in their size, density, and lipid composition and also contain rat apo E; but they differ in that they lack apo B48. Earlier studies, however, have demonstrated that this type of model particle without apo B48 is cleared from the blood and metabolized in vivo in a similar way to physiologic lipoproteins, and also mimics their effects in cultured cells in vitro [29,41,44,45].

Our previous work has shown that CMR from rats given a fat meal of palm, olive, corn, or fish oil contain a wide range of fatty acids, but are particularly enriched in SFA, MUFA, n-6 PUFA, and n-3 PUFA, respectively, reflecting the fatty acid composition of the oil fed [38]; and these differences were found to influence the hepatic uptake and processing of the particles [40,46,47]. To mimic the physiologic situation as closely as possible, in this study, we used CRLPs containing TG derived from palm, olive, or corn oils so that the particles were enriched in the fatty acids predominating in the oils but, as in vivo, also contained a complex mixture of other fatty acids. In earlier work in our laboratory [39], we have shown that the fatty acid composition of CRLPs containing TG from palm, olive, and corn oil is similar to that of the parent oils and also that of rat physiologic CMR derived from them [38]; thus, the palm, olive, or corn CRLPs were enriched in SFA (mainly palmitic acid, 16:0), MUFA (mainly oleic acid, 18:1n-9), and n-6 PUFA (mainly linoleic acid, 18:2n-6), respectively. There were no significant differences, however, in the TG or TC content, the TG/TC ratio, or the apo E content (Table 2, Fig. 1) of the 3 types of particles. The differences in their effects on VLDL secretion observed in the present study, therefore, can be attributed to the variation in their fatty acid composition.

Previous investigations of the effects of dietary fatty acids on hepatic VLDL secretion by the direct addition of NEFA to hepatocyte cultures using primary rat hepatocytes or the human liver cell line HepG2 have found consistently that it is suppressed by exposure of cells to n-3 PUFA and stimulated by oleic acid, although information about the effects of SFA and n-6 PUFA is more limited and conflicting [48-51]. Dietary fatty acids, however, are delivered to the liver in CMR rather than as NEFA; and evidence suggests that these different delivery routes influence VLDL secretion in different ways [21,28]. For this reason, in the present study, we used CRLPs and rat hepatocytes to compare the direct effects of SFA, MUFA, and n-6 PUFA carried in CMR on VLDL secretion.

It is clear from our results that the secretion of TG into the medium by the cells was increased by palm or corn, but not olive, CRLPs and thus was lower in hepatocytes exposed to olive CRLPs as compared with palm or corn CRLPs (Fig. 2). Cholesterol secretion, on the other hand, was not increased by any of the CRLP types, although there was a tendency toward a decrease in cells treated with olive as compared with palm or corn CRLPs. The lipid content of the *d* less than 1.050–g/mL fraction, which contained most of the TG and cholesterol secreted in VLDL and any intermediate-density lipoprotein and LDL formed from it by lipases produced by the cells (Fig. 2), also showed a similar pattern, indicating that lipid secretion in VLDL is increased in liver cells exposed to CMR enriched in SFA and n-6 PUFA as compared with MUFA.

In previous work, we have shown that CMR enriched in SFA, MUFA, or n-6 PUFA are bound and internalized by rat hepatocytes at similar rates, whereas n-3 PUFA are internalized more rapidly [47]; thus, the decreased secretion of lipid in VLDL caused by olive CRLPs reported here is unlikely to be due to a lower rate of uptake of the particles. However, in earlier experiments with the perfused liver, we have also found that TGs from CMR high in MUFA are used for oxidation at approximately 3 times the rate of those high in SFA or n-6 PUFA [46]. Thus, when the CRLPs are enriched in MUFA as compared with SFA or n-6 PUFA, the TG available for VLDL secretion by the hepatocytes may be decreased.

In a number of studies, treatment of hepatocytes with nonesterified MUFA (oleic acid, 18:1n-9) has consistently been found to increase VLDL TG and cholesterol secretion in rat hepatocytes and the human liver cell line HepG2 [48-52]. However, these studies used a higher dose (0.8-1 mmol/L oleic acid) than our experiments (0.3 mmol/L CRLP TG); and Homan et al [53] did not find any increase in TG mass secretion with 0.3 mmol/L oleic acid in HepG2 cells. Nonesterified palmitic (SFA, 16:0) and linoleic (PUFA, 18:2n-6) acids have also been reported to increase VLDL TG and cholesterol secretion in rat or chick hepatocytes [24,52,54]; but comparisons between fatty acids have given conflicting results, with Rustan and colleagues [24,52] finding no difference in the effects of palmitic, oleic, or linoleic acids on the output of newly synthesized TG or cholesterol by rat hepatocytes, whereas Wong and Nestel [49] reported that linoleic acid (PUFA, 18:2 n-6) decreased TG secretion in HepG2 cells as compared with oleic acid. Our results, however, provide new evidence to suggest that MUFA as compared with SFA or n-6 PUFA decrease the secretion of VLDL lipid by hepatocytes and that this effect occurs directly on delivery of the fats to the liver in CMR.

The finding that the TG content of the hepatocytes was unaffected by the 3 types of CRLPs tested, together with our earlier work showing that oleate from TG delivered in CMR is oxidized rapidly [46], suggests that remnant TG is used in preference to endogenous TG for VLDL secretion. If endogenous TG were an important source, the TG content of hepatocytes treated with olive CRLPs would be expected to rise, as no increase in VLDL secretion was observed in these conditions. In agreement with our previous study [28], corn oil CRLPs had no effect on cell cholesterol concentrations. However, there was a small (about 9%-12%) but significant decrease in the TC content of cells treated with palm or olive CRLPs as compared with corn CRLPs or untreated controls (Fig. 3) that was not due to increased secretion of cholesterol in VLDL because this was not changed. Earlier studies, however, have found that dietary SFA decreases hepatic cholesterol levels as compared with n-6 PUFA [55-58]. The mechanisms of this effect are not clear, but Smit et al [58] have shown that hepatic plasma membranes from rats fed n-6 PUFA contained more cholesterol than those from animals fed SFA or MUFA. We have also found in previous work that the activity and expression of cholesterol 7*α* hydroxylase, the rate-limiting enzyme for the synthesis of bile acids from cholesterol, are increased in rats fed olive as compared with corn or palm oil [55]. Thus, the lower levels of cholesterol in the cells treated with olive CRLPs in the present study may be due to stimulation of the bile acid synthesis pathway.

Apolipoprotein B is an integral protein in VLDL [18] and is essential for its assembly and secretion. It exists in 2 forms: the full-length protein (apo B100) and its N-terminal 48% (apo B48). In many species, including humans, hepatic VLDL contains only apo B100; but rat hepatic VLDL contains both apo B100 and apo B48 [59]. Previous studies with NEFA have generally found that secretion of both apo B100 and apo B48 in rat hepatocytes is suppressed by n-3 PUFA but is unaffected by oleic acid [27,51,60,61]. When rats are fed fish oil in the diet, however, only apo B48 secretion is inhibited [27]. Thus, the route of delivery of fatty acids to the liver is important in determining their effects on hepatic apo B secretion. Fatty acids from the diet reach the liver in CMR; and in agreement with the findings of Brown et al [27], our previous work has shown that the secretion of apo B48, but not apo B100, by rat hepatocytes was decreased by CMR enriched in n-3 PUFA, but CMR enriched in n-6 PUFA had no significant effects [28]. In the present study, the secretion of apo B48 by rat hepatocytes was not affected by exposure to palm, olive, or corn CRLPs (Fig. 4). Although we were able to detect apo B48 in the whole medium, we found that levels in the *d* less than 1.05–g/mL fraction were too low to measure accurately. Thus, some of the apo B48–containing particles secreted must have a *d* greater than 1.05g/mL. This is in agreement with previous studies, which have also found apo B48 in higher-density lipoprotein fractions secreted by rat hepatocytes [62,63]. Palacios et al [63] attributed this to the secretion of small, lipid-poor VLDL. Other contributory factors might include lipolysis of some secreted VLDL by lipases such as hepatic lipase produced by the liver cells, or the release of smaller particles bound at the cell surface by apo E. The amounts of apo B100 secreted by rat liver cells were too low to measure accurately in the current investigation. However, in previous studies, apo B100 secretion by rat hepatocytes has proven unresponsive to dietary fatty acids except when incubated with n-3 PUFA directly [25,27,28,51,60]. Because each VLDL particle contains 1 molecule of apo B [18], the finding that the decreased secretion of TG caused by olive as compared with palm or corn CRLPs is not accompanied by decreased secretion of apo B48 suggests that uptake of CMR enriched in MUFA rather than SFA or n-6 PUFA results in the secretion of smaller VLDL particles. It is possible, however, that the smaller particles may result from differential postsecretory lipolysis or oxidation, rather than initial secretion. The production of large VLDL has been associated with increased plasma levels of small dense LDL, which are believed to be more atherogenic than larger, more buoyant LDL because they are more susceptible to oxidation and are more likely to enter the artery wall [64]. Thus, the promotion of the secretion of smaller VLDL by CMR enriched in MUFA may be a contributory factor in the beneficial effects of olive oil.

One apo B gene codes for both apo B100 and apo B48. Messenger RNA for the full-length protein is edited posttranscriptionally by conversion of codon 2153 into a stop signal [65] to form the apo B48 transcript. In the present study, the expression of mRNA for apo B in rat hepatocytes was not changed by any of the CRLP types tested after 5 hours of incubation; but after 16 hours, there was a modest decrease of 30% to 40% with olive and corn CRLPs (Fig. 5A). These results are consistent with the finding that there was no change in apo B48 secretion after 5 hours of exposure to the particles, but suggest that, in the longer term, there may be a down-regulation of the transcription of apo B mRNA by MUFA and n-6 PUFA delivered to the liver in CMR.

The expression of mRNA for the MTP (essential for VLDL assembly) and for enzymes involved in the regulation of hepatic TG (mGPAT) and CE (ACAT-2) synthesis in the hepatocytes was unaffected by CRLPs, regardless of their fatty acid composition. These results are in agreement with our previous work showing no effect of rat corn CMR on MTP mRNA expression [66] and suggest that modulation of transcription of these genes is not involved in the effects of fatty acids delivered to the liver in CMR on VLDL secretion. The CRLPs also had no effect of the expression of mRNA for HMG-CoA reductase, the rate-limiting enzyme in cholesterol synthesis, after 5 hours of incubation with the cells; but after 16 hours of incubation, all CRLP types tended to increase mRNA levels for this enzyme, although this change was significant only with palm CRLPs (Fig. 5B). These findings suggest that cholesterol synthesis may be up-regulated by CRLPs containing SFA, possibly in response to the fall in cellular cholesterol levels (Fig. 3).

In conclusion, the results of this study demonstrate that CMR enriched in SFA or n-6 PUFA increase the secretion of TG in VLDL, whereas CMR enriched in MUFA have no effect. Secretion of apo B48, however, was not changed in all cases, suggesting that the rise in VLDL TG observed with particles enriched in SFA and n-6 PUFA as compared with MUFA is due to the secretion of larger particles. Because large VLDLs are associated with increases in plasma atherogenic small dense LDL, these findings suggest that dietary MUFA may be beneficial in this respect. Together with our earlier work showing that the secretion of VLDL is decreased by CMR enriched in n-3 PUFA [28], these findings indicate that different dietary fats have differential effects on VLDL secretion directly when delivered to the liver in CMR.

## Figures and Tables

**Table 1:** Primer sequences and product sizes for qPCR

Gene		Sequences forward and reverse primers (5′-3′)	Product size (base pair)
apo B	Forward	AGAAAGAGAACAAAGCAGAGATTGTGG	170
	Reverse	CATGCTCCATTCTCACATGTTTA	
MTP	Forward	AGT GAT TTG ATG TCC AAA ATG CT	160
	Reverse	AAC CAG AAA TGT CAA TGG CTA GA	
mGPAT	Forward	CCA GAC ATT TTA CCA GGT TTG TC	177
	Reverse	CTG TCC TCA TCT TCT TCG TCA CT	
HMG-CoA reductase	Forward	CAA AGA CAA TCC TGG AGA AAA TG	166
	Reverse	TGTTCCCTGCAGATCTTGTAA AT	
ACAT-2	Forward	ACATATGGAGGCTGTGAAGACAC	164
	Reverse	GGTGTAACTCTTGGGTCT TCC TT	
GAPDH	Forward	GATGACATCAAGAAGGTGGTG A	206
	Reverse	ACCCTGTTGCTG TAG CCATATT	

**Table 2:** Lipid content of CRLPs derived from different oils

CRLP type	TG (*μ*mol/mL)	TC (*μ*mol/mL)	UC (*μ*mol/mL)	CE (*μ*mol/mL)	TG/TC
Palm	7.8 ± 1.2	0.8 ± 0.2	0.4 ± 0.1	0.4 ± 0.1	11.2 ± 1.0
Olive	9.2 ± 1.5	0.9 ± 0.2	0.5 ± 0.2	0.4 ± 0.1	11.1 ± 0.8
Corn	8.0 ± 1.1	0.8 ± 0.1	0.5 ± 0.2	0.3 ± 0.1	10.7 ± 0.9

Chylomicron remnant–like particles containing TG from palm, olive, or corn oils were prepared as described in “Materials and methods”; and TG, TC, UC, and CE contents were measured. Data shown are the mean ± SEM from 9 separate preparations.
